# The role of osteocalcin in regulation of glycolipid metabolism and muscle function in children with osteogenesis imperfecta

**DOI:** 10.3389/fendo.2022.898645

**Published:** 2022-08-02

**Authors:** Wen-bin Zheng, Jing Hu, Di-Chen Zhao, Bing-Na Zhou, Ou Wang, Yan Jiang, Wei-Bo Xia, Xiao-ping Xing, Mei Li

**Affiliations:** ^1^ Department of Endocrinology, National Health Commission Key Laboratory of Endocrinology, Peking Union Medical College Hospital, Chinese Academy of Medical Sciences and Peking Union Medical College, Beijing, China; ^2^ Department of Endocrinology, Union Hospital, Tongji Medical College, Huazhong University of Science and Technology, Wuhan, China

**Keywords:** osteocalcin, undercarboxylated osteocalcin, glycolipid metabolism, muscle function, osteogenesis imperfect

## Abstract

**Objective:**

Osteoblasts are discovered to secrete hormones with endocrine effects on metabolism, and osteocalcin (OC) is the most abundant non-collagenous protein in bone. We investigate the relationship between serum OC levels and glycolipid metabolism and muscle function in children with osteogenesis imperfecta (OI).

**Methods:**

A total of 225 children with OI and 80 healthy controls matched in age and gender were included in this single center study. Serum levels of fasting blood glucose (FBG), triglyceride (TG), total cholesterol (TC), low- and high-density lipoprotein cholesterol (LDL-C, HDL-C) were measured by automated analyzers. Serum levels of fasting insulin (FINS) were measured using an automated electrochemiluminescence system. Serum levels of OC and undercarboxylated osteocalcin (ucOC) were measured by enzyme-linked immunosorbent assay. Grip strength and timed-up-and-go (TUG) test were measured. Bone mineral density (BMD) and body composition were measured using dual-energy X-ray absorptiometry.

**Results:**

OI patients had significantly higher body mass index (BMI), FBG, and HOMA-IR, but lower HDL-C levels, lower grip strength and longer TUG than control group (all *P*<0.05). Serum OC, ucOC levels, and ucOC/OC in OI type III patients were significantly lower than those in OI patients with type I and IV. Serum levels of OC, ucOC, and ucOC/OC were negatively correlated to BMI, FBG, insulin levels, and HOMA-IR (all *P*<0.05). The ratio of ucOC/OC was positively correlated to grip strength (r=0.512, *P*=0.036), lean mass percentage (%LM) of the total body and limbs, and negatively correlated to fat mass percentage (%FM) of the total body, %FM and fat mass index (FMI) of the trunk (all *P*<0.05).

**Conclusions:**

Obesity, glucolipid metabolic abnormalities, and reduced grip strength were common in children with OI. Circulating osteocalcin and ucOC may play an important role in the regulation of glucose metabolism, as well as the muscle function of children with OI.

## Introduction

Bone is a specialized connective tissue that provides mechanical support for organs, acts as an anchor site for muscles, protects vital organs, houses the hematopoietic bone marrow, and maintains mineral homeostasis. Bone is a dynamic tissue that is formed by osteoblasts and resorbed by osteoclasts in a continuous remodeling cycle. Recently, osteoblasts are discovered to secrete several hormones with endocrine effects on metabolism. These endocrine factors include osteocalcin, lipocalin and sclerostin, and so on (1). Osteocalcin, also referred as bone γ-carboxyglutamic acid protein, is the most abundant non-collagenous protein in bone matrix and is produced almost exclusively by the osteoblasts (2, 3). In osteoblasts, osteocalcin undergoes carboxylation at three glutamate residues (17, 21, and 24) by γ-glutamyl carboxylase after protein translation at the endoplasmic reticulum. The acidic environment generated during bone resorption processes promotes decarboxylation of c-carboxylated osteocalcin trapped in the bone matrix to undercarboxylated osteocalcin, decreasing its affinity for hydroxyapatite and therefore promoting its release into the circulation, which formed its function as a hormone (4). OC has previously been used as a convenient biomarker of bone formation. Interestingly, the study demonstrated OC^−/−^ mice were obese, with glucose-intolerance due to decreased insulin production and β-cell proliferation, and were insulin-resistant in comparison to wild mice (5). In 2016, a second paradigm has emerged that OC regulated skeletal muscle metabolism and physiology (6). However, under the condition of bone disease, it is unclear whether the production of OC is abnormal and has an impact on the patient’s glycolipid metabolism and muscle function.

Osteogenesis imperfecta (OI) is a rare genetic disease characterized by decreased bone mineral density (BMD), recurrent bone fractures, and progressive bone deformity. Mutations in *COL1A1* and *COL1A2* are the main cause of OI, and the other patients may carry gene mutations related to the abnormality of modifying enzymes, chaperone proteins of type I collagen, or of osteoblasts functions (7, 8). Recently, obesity, overweight, and impaired muscle function have caused concern as common disorders in OI patients (9), which will further increase the risk of diabetes, hyperlipidemia, falls, and fractures. However, the underlying mechanisms of these metabolic disorders and muscle dysfunction in OI patients are unknown. Whether OC is involved in metabolic disorders and muscle dysfunction in OI patients is worth investigating.

Therefore, the objective of this study is to evaluate glycolipid metabolism and muscle function of children with OI and to investigate their relationship to serum levels of OC and ucOC.

## Materials and methods

### Study design and participants

This was a single-center cross-sectional study conducted in endocrinology department of Peking Union Medical College Hospital (PUMCH) from January 2018 to April 2021. Patients with OI less than 18 years old and age- and gender- matched normal controls were included. The patients were diagnosed as OI if they met the criteria: Patients had a history of more than one fracture under minor trauma, and with an age- and sex-adjusted BMD Z-score less than or equal to -1.0 at lumbar spine (LS) or femoral neck (FN); or patients with BMD Z-scores ≤ -2.0 at LS or FN; and then the pathogenic mutation was found in these patients (10, 11). Patients with fracture history within the recent 6 months prior to enrollment were excluded to reduce the influence of recent fracture on the outcomes. Participants were excluded if they had other inherited or metabolic bone disease, a previous treatment history of bisphosphonates, or had received treatment that could affect the glycolipid metabolism or muscle function, or with obvious abnormal liver and kidney function.

Patients with OI were classified into subtypes according to Sillence classification and clinical characters: type I, mild phenotype; type II, perinatally lethal; type III, a severe form with progressive deformity; type IV, moderate severity; and type V, characterized by calcification of the hyperplastic callus formation, forearm interosseous membrane and radial head dislocation.

The study was approved by the Scientific Ethics Committee of PUMCH. The legal guardians of OI patients and normal controls provided written informed consents before they participated in this study.

### Basic information collection

Medical history and family history was collected in detail. Height and weight of the patients were measured by Harpenden stadiometer (Seritex Inc., East Rutherford, NJ, USA), and the age- and gender-specific Z scores were calculated according to the normal reference of Chinese children (12). Body mass index (BMI) was calculated as weight(kg) divided by the square of height(m^2^). Overweight and obesity were defined as BMI higher than the 85th and 95th percentile of the reference of Chinese normal children (13).

### Measurements of serum levels of OC and glycolipid metabolic parameters

Blood samples were collected after an overnight fast. Serum levels of OC and ucOC were measured by enzyme-linked immunosorbent assay (ELISA, Takara Bio Inc, Japan) following the manufacturer’s instructions, with the detection range of OC and ucOC as 0.5-16.0 ng/ml and 0.25-8.0 ng/ml, respectively. The intra-assay coefficients of variation (CV) were 3.0%-4.8% and 4.4%-6.7% for OC and ucOC measurement, respectively. The inter-assay CVs were 0.7%-2.4% and 5.7%-9.9% for OC and ucOC detection, respectively.

Serum levels of fasting blood glucose, triglyceride, total cholesterol, low-density lipoprotein cholesterol, high-density lipoprotein cholesterol, calcium (Ca), phosphate (P), alkaline phosphatase (ALP, a bone formation marker), alanine aminotransferase (ALT) and creatinine (Cr) were measured by automated analyzers (ADVIA1800, Siemens, Germany). Serum levels of fasting insulin (FINS), beta cross-linked carboxy-terminal telopeptide of type I collagen (β-CTX, a bone resorption marker), 25-hydroxyvitamin D (25OHD), and intact parathyroid hormone (PTH) were measured using an automated electrochemiluminescence system (E170; Roche Diagnostics, Switzerland). Values of insulin sensitivity/resistance were calculated as Homeostasis model assessment insulin resistance (HOMA-IR), which equaled to FBG (mmol/L) × FINS (μU/mL)/22.5. Homeostasis model assessment islet beta-cell function (HOMA-β) was equal to 20×FINS (μU/mL)/(FPG, mmol/L)-3.5) (%). As renal function was a possible influence factor of OC (14), we used the Schwartz equation to estimate glomerular filtration rate (eGFR) (15). Parameters of glycolipid metabolism were also measured in the normal control group.

### Measurement of BMD and body composition

Total-body composition, BMD at lumbar spine 2–4 (LS), femoral neck (FN) and total hip of OI patients were measured by dual-energy X-ray absorptiometry (DXA, Lunar Prodigy Advance, GE Healthcare, USA). BMD Z scores of LS and femoral neck were calculated based on reference data of BMD in Chinese and Asian children (16, 17). Each scan of DXA was reviewed by a radiologist, and the CVs of DXA measurement was 0.8% to 1.0%. Body fat mass percentage (%FM), lean mass percentage (%LM) were calculated by total fat mass, lean mass divided by the sum of bone, lean and fat mass, respectively. Fat mass index (FMI) and lean mass index (LMI) were also calculated as body fat mass (kg), lean mass (kg) divided by square of height (m^2^). Appendicular mass was calculated as the sum of upper and lower limb mass. For males, slight-moderate or severe increased body fat was defined as body fat mass percentage more than 20% or more than 25%, respectively. For females, slight-moderate or severe increased body fat was defined as body fat mass percentage more than 30% or more than 35%, respectively (18).

### Measurement of muscle function

Grip strength of the dominant hand was measured by a handheld dynamometer (Hand Grip Dynamometer, FEINECE. Inc. China), and the highest of three attempts was recorded. Physical function was measured by the timed-up-and-go (TUG) test, of which patients were timed to stand from the chair, walk 3 meters, turn, and then return to the seated position in the chair (19).

### Detection of genetic mutation of OI

Genetic mutations of OI were identified by a targeted next-generation sequencing (NGS) panel (Illumina HiSeq2000 platform, Illumina, Inc., San Diego, CA, USA) which was previously described in detail (20), and candidate genes of OI were covered in this panel, including *COL1A1*, *COL1A2*, *IFITM5*, *SERPINF1*, *CRTAP*, *SERPINH1*, *FKBP10*, *SP7*, *BMP1*, *TMEM38B*, *PLOD2*, *P3H1*, *P4HB*, *PPIB*, *SEC24D*, *SPARC*, *WNT1*, *PLS3*, *CREB3L1 and MBTPS2* (20). The detected gene mutation of NGS was further confirmed by polymerase chain reaction (PCR) and Sanger sequencing.

### Statistical analysis

Continuous data of normal distribution (including age, height, weight, BMI, FBG, TC, TG, HDL-C, LDL-C, OC, ucOC, Ca, P, ALP, β-CTX, Cr, eGFR, and BMD) were expressed as mean ± standard deviation (SD). Abnormal distribution (including times of fracture, insulin, 25OHD, PTH and ALT levels, HOMA-IR, and HOMA-β) were presented as median (quartiles). Categorical data were expressed as the number and percentage (%). The independent sample t-test and the analysis of variance (ANOVA) were utilized to compare continuous data of normal distribution among different clinical or genetic subgroups. Continuous data of abnormal distribution for two groups and more than two groups were analyzed by the Mann-Whitney U-test and Kruskal-Wallis test, respectively. The Chi-squared test and the Fisher test were used to analyze categorical variables. Relationships between OC or ucOC levels and glycolipid metabolic parameters or muscle function were analyzed using the Spearman correlation. Multiple linear regression models were used for adjustments of age, gender, 25OHD, eGFR, ambulatory status, and clinical classifications.

The SPSS software version 20.0 (SPSS, Inc., Chicago, IL, USA) was used to perform all statistical analyses. Statistical significance was considered if the two-tailed *P* value was less than 0.05.

## Results

### Clinical characteristics of OI patients and controls

A total of 225 children with OI were included in this study, with mean age of 8.0 ± 4.7 years. Eighty healthy children were included as controls, with mean age of 8.0 ± 3.5 years. BMI of OI patients was 18.7 ± 4.0 kg/m^2^, which were higher than that of control group(*P*=0.003). There were 45 (20.0%) OI patients with obesity, and the percentage was higher than the control group (*P*=0.043). OI patients had significantly higher FBG levels and HOMA-IR, but lower HDL-C levels than the control group (all *P*<0.05). The serum levels of OC in OI patients were (24.56 ± 11.51) ng/ml, which were lower than the control group (*P*=0.040). The serum of ucOC and the ratio of ucOC/OC had no significant difference between the OI patients and control group (**Table 1**). In addition, no obvious differences in serum levels of OC, ucOC, and ucOC/OC were found between male and female patients with OI.

**Table 1:** General characteristics of OI patients and control group.

	OI patients	Healthy control	*P* value
	(n = 225)	(n = 80)	
Male, n (%)	151 (67.1%)	44 (55.0%)	0.051
Age (y)	8.0±4.7	8.0±3.5	0.893
Height (cm)	119.6±27.9	130.5±20.0	**<0.001**
Height Z-score	-1.6±2.6	0.5±1.3	**<0.001**
Weight (kg)	29.0±16.1	31.2±12.2	0.213
Weight Z-score	-0.1±1.5	0.9±1.1	**<0.001**
BMI (kg/m^2^)	18.7±4.0	17.6±2.4	**0.003**
Overweight, n (%)	12 (5.3%)	4 (5.0%)	0.759
Obese, n (%)	45 (20.0%)	8 (10.0%)	**0.043**
FBG (mmol/L)	5.06±0.40	4.93±0.48	**0.020**
Insulin (μIU/mL)	9.00 (5.25, 13.95)	7.20 (4.75, 12.70)	0.072
HOMA-IR	1.96 (1.12, 3.27)	1.50 (1.00, 2.66)	**0.031**
HOMA-β (%)	117.50 (77.71, 181.99)	112.86 (65.56, 165.00)	0.252
TC (mmol/L)	4.21±0.79	4.35±0.81	0.223
TG (mmol/L)	0.82±0.45	0.77±0.49	0.445
HDL-C (mmol/L)	1.04±0.26	1.45±0.29	**<0.001**
LDL-C (mmol/L)	2.24±0.57	2.27±0.61	0.682
ucOC (ng/ml)	11.14±8.27	13.16±7.37	0.059
OC (ng/ml)	24.56±11.51	28.15±11.54	**0.040**
ucOC/OC	0.43±0.19	0.44±0.20	0.681
ALT (IU/L)	14.0 (11.0, 18.0)	13.0 (11.0, 17.5)	0.608
Cr (μmol/L)	35.7±12.2	46.8±12.9	**<0.001**
eGFR (ml/min/1.73m^2^)	170.6±43.0	151.9±27.5	**<0.001**

OI, osteogenesis imperfecta; BMI, body mass index; FBG, fasting blood glucose; HOMA-IR, homeostasis model assessment insulin resistance; HOMA-β, homeostasis model assessment islet beta cell function; TG, triglyceride; TC, total cholesterol; HDL-C, high density lipoprotein cholesterol; LDL-C, low density lipoprotein cholesterol; OC, osteocalcin; ucOC, undercarboxylated osteocalcin; ALT, alanine aminotransferase; Cr, creatinine; eGFR, estimated glomerular filtration rate.

### Levels of OC and glycolipid metabolic parameters among different clinical subgroups of OI

There were 96, 40, 81, and 8 patients classified as type I, type III, type IV, and type V of OI (**Table 2**). Since OI type II was perinatal lethal, no patients with OI type II were included in this study. Patients with OI type III had lower height and weight than patients with OI type I and IV, but higher BMI than type IV OI patients (*P*<0.05). Overweight was found in 8 (8.3%), 3 (7.5%), and 1 (1.2%) patients with type I, type III and type IV OI, and obesity existed in 16 (16.7%), 11 (27.5%) and 14 (17.3%) of the above subgroups patients with OI, respectively. Serum levels of FBG were significantly higher in OI type III patients than in OI type I and type IV patients, but HOMA-IR, HOMA-β, TC, LDL-C, HDL-C, and TG were similar among OI type I, III and IV groups. Serum OC, ucOC levels, and ucOC/OC in OI type III patients were significantly lower than those in OI patients with type I and IV (**Table 2**).

**Table 2:** Clinical characteristics of OI patients with different clinical classifications.

	Total OI patients	OI-type I	OI-type III	OI-type IV	*P* value
	(n = 225)	(n = 96)	(n = 40)	(n = 81)	
Male, n (%)	151 (67.1%)	64 (66.7%)	22 (55.0%)^c^	62 (76.5%)^c^	0.052
Age (y)	8.0 ± 4.7	8.8 ± 4.5	7.8 ± 5.1	7.7 ± 4.7	0.193
Height (cm)	119.6 ± 27.9	128.3 ± 27.4^a,b^	103.9 ± 25.6^a,c^	118.2 ± 26.0^b,c^	**<0.001**
Height Z-score	-1.6 ± 2.6	-0.9 ± 1.9^a^	-3.9 ± 3.7^a,c^	-1.5 ± 2.1^c^	**0.001**
Weight (kg)	29.0 ± 16.1	33.7 ± 16.9^a,b^	22.9 ± 12.6^a^	27.8 ± 15.8^b^	0.087
Weight Z-score	-0.1 ± 1.5	0.3 ± 1.5^a,b^	-1.4 ± 1.8^a,c^	-0.3 ± 1.1^b,c^	**<0.001**
BMI (kg/m^2^)	18.7 ± 4.0	19.0 ± 3.7	19.8 ± 4.6^c^	18.2 ± 3.6^c^	**0.047**
Overweight, n (%)	12 (5.3%)	8 (8.3%)^b^	3 (7.5%)	1 (1.2%)^b^	0.100
Obese, n (%)	45 (20.0%)	16 (16.7%)	11 (27.5%)	14 (17.3%)	0.304
Nonambulatory, n (%)	85 (37.8%)	16 (16.7%)^a,b^	32 (80.0%)^a,c^	35 (43.2%)^b,c^	**<0.001**
Times of fracture	4.0 (3.0, 6.5)	3.0 (2.0, 5.0)^a^	10.0 (4.3, 20.0)^a,c^	5.0 (3.3, 6.0)^c^	**<0.001**
Ca (mmol/L)	2.46 ± 0.20	2.47 ± 0.09	2.48 ± 0.11	2.44 ± 0.30	0.536
P (mmol/L)	1.66 ± 0.20	1.68 ± 0.20	1.66 ± 0.23	1.64 ± 0.19	0.489
ALP (U/L)	286.6 ± 106.0	298.0 ± 104.6^a^	253.9 ± 110.5^a^	283.5 ± 95.2	0.078
β-CTX (ng/ml)	0.85 ± 0.35	0.90 ± 0.36^a,c^	0.64 ± 0.30^a^	0.84 ± 0.33c	**0.023**
25OHD (ng/ml)	22.3 (15.6, 30.8)	20.0 (14.1, 31.0)	25.5 (16.4, 41.2)	22.8 (15.5, 30.1)	0.658
PTH (ng/ml)	21.4 (13.9, 31.2)	23.4 (17.6, 33.0)^a^	18.4 (12.5, 25.6)^a^	18.6 (11.0, 32.4)	0.072
ALT (U/L)	14.0 (11.0, 18.0)	14.0 (11.3, 19.0)^a^	11.0 (9.0, 16.0)^a^	14.0 (11.0, 18.5)	0.097
Cr (μmol/L)	35.7 ± 12.2	38.3 ± 12.1^a,b^	33.6 ± 11.4^a^	34.2 ± 12.6^b^	**0.039**
eGFR (ml/min/1.73m^2^)	170.6 ± 43.0	172.4 ± 37.1^a^	156.1 ± 47.4^a,c^	176.8 ± 44.9^c^	**0.013**
FBG (mmol/L)	5.06 ± 0.40	5.01 ± 0.35^a^	5.27 ± 0.41^a,c^	5.03 ± 0.39^c^	**0.002**
Insulin (μIU/mL)	9.00 (5.25, 13.95)	8.00 (4.55, 13.90)	9.00 (7.40, 12.80)	10.00 (5.30, 17.70)	0.459
HOMA-IR	1.96 (1.12, 3.27)	1.86 (1.04, 3.24)	2.08 (1.68, 3.22)	2.31 (1.18, 4.01)	0.466
HOMA-β (%)	117.50 (77.71, 181.99)	111.57 (66.68, 177.27)	111.43 (92.50, 156.56)	140.41 (75.38, 248.03)	0.263
TC (mmol/L)	4.21 ± 0.79	4.14 ± 0.86	4.11 ± 0.65	4.31 ± 0.78	0.878
TG (mmol/L)	0.82 ± 0.45	0.82 ± 0.48	0.80 ± 0.39	0.85 ± 0.46	0.334
HDL-C (mmol/L)	1.04 ± 0.26	1.03 ± 0.22	1.07 ± 0.25	1.04 ± 0.26	0.812
LDL-C (mmol/L)	2.24 ± 0.57	2.21 ± 0.60	2.12 ± 0.41	2.33 ± 0.60	0.177
ucOC (ng/ml)	11.14 ± 8.27	11.41 ± 9.84^a^	7.36 ± 6.55^a,c^	12.39 ± 8.74^c^	**0.001**
OC (ng/ml)	24.56 ± 11.51	26.08 ± 10.66^a^	17.47 ± 9.03^a,c^	26.01 ± 12.66^c^	**<0.001**
ucOC/OC	0.43 ± 0.19	0.42 ± 0.18	0.36 ± 0.19^c^	0.46 ± 0.18^c^	**0.025**
LS BMD (g/cm^2^)	0.465 ± 0.198	0.540 ± 0.196^a,b^	0.358 ± 0.182^a^,c	0.443 ± 0.178^b^,c	**<0.001**
LS BMD Z-score	-2.2 ± 2.0	-1.5 ± 1.8^a,b^	-3.4 ± 2.1^a^,c	-2.6 ± 1.9^b,c^	**<0.001**
FN BMD (g/cm^2^)	0.426 ± 0.189	0.515 ± 0.185^a,b^	0.296 ± 0.196^a,c^	0.392 ± 0.146^b,c^	**<0.001**
FN BMD Z-score	-3.7 ± 2.5	-2.7 ± 1.9^a,b^	-5.4 ± 2.9^a,c^	-4.1 ± 1.9^b,c^	**<0.001**
Troch BMD (g/cm^2^)	0.347 ± 0.191	0.415 ± 0.195^a,b^	0.265 ± 0.188^a^	0.314 ± 0.161^b^	**<0.001**
TH BMD (g/cm^2^)	0.469 ± 0.207	0.550 ± 0.213^a,b^	0.363 ± 0.196^a^	0.430 ± 0.162^b^	**<0.001**

OI, osteogenesis imperfecta; BMI, body mass index; Ca, calcium; P, phosphate; ALP, alkaline phosphatase; β-CTX, β cross-linked carboxy-terminal telopeptide of type I; 25OHD, 25-hydroxyvitamin D; PTH, parathyroid hormone; FBG, fasting blood glucose; HOMA-IR, homeostasis model assessment insulin resistance; HOMA-β, homeostasis model assessment islet beta cell function; TG, triglyceride; TC, total cholesterol; HDL-C, high density lipoprotein cholesterol; LDL-C, low density lipoprotein cholesterol; OC, osteocalcin; ucOC, undercarboxylated osteocalcin; ALT, alanine aminotransferase; Cr, creatinine; eGFR, estimated glomerular filtration rate. LS, lumbar spine; FN, femoral neck; Troch, trochanter; TH, total hip; BMD, bone mass density.

Bold values indicate that there was a significant difference among 3 groups. ^a^ *P* < 0.05 for comparison between OI-I and OI-III; ^b^ *P* < 0.05 for comparison between OI-I and OI-IV; ^c^ *P* < 0.05 for comparison between OI-III and OI-IV.

### Levels of OC and glycolipid metabolic parameters among different genotypes of OI

The mutated genes identified in OI patients included *COL1A1* (n=126, 56.0%), *COL1A2* (n=66, 29.3%), *IFITM5* (n=8, 3.6%), *FKBP10* (n=5, 2.2%), *WNT1* (n=5, 2.2%), *PLS3* (n=3, 1.3%), *TMEM38B* (n=2, 0.9%), *PLOD2* (n=2, 0.9%), *SERPINF1* (n=2, 0.9%), *BMP1* (n=2, 0.9%), *SERPINH1* (n=1, 0.4%), *CRTAP* (n=1, 0.4%), and *P3H1* (n=1, 0.4%).

According to the pattern of pathogenic gene mutation, OI patients were merged and classified into *COL1A1*, *COL1A2*, *IFITM5* and the autosomal recessive (AR) gene mutations group. Patients with *IFITM5* mutation had significantly lower BMI than AR group. There was no significant difference in the percentage of overweight or obesity, serum levels of FBG, insulin, HOMA-IR, HOMA-β, TG, TC, LDL-C, HDL-C, OC, ucOC and ucOC/OC among the four subgroups of genotypes (**Supplementary Table 1**).

### Associations between OC levels with metabolic parameters

**Figure 1:**
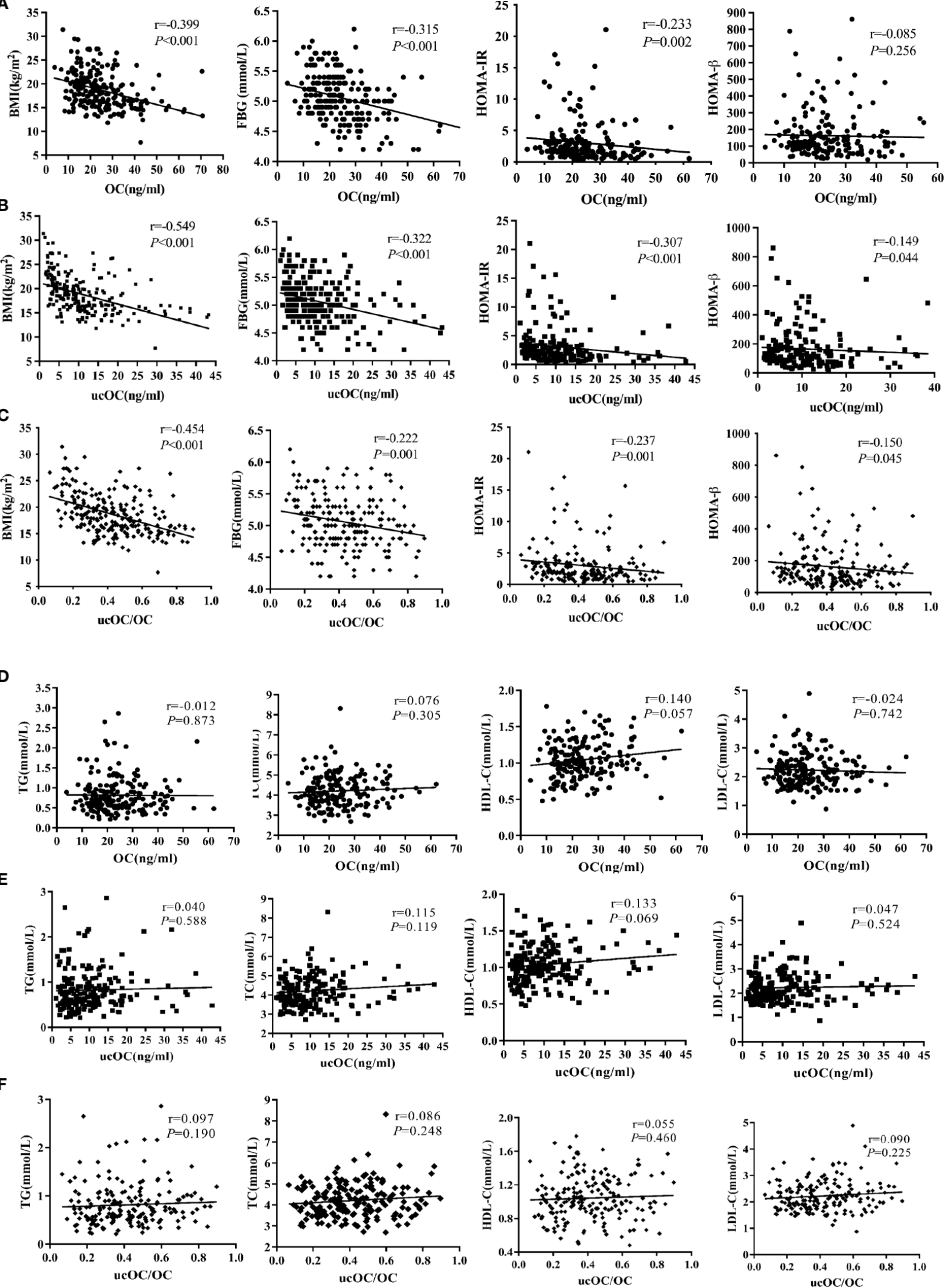
Associations between serum levels of OC, ucOC and ucOC/OC with glycolipid metabolic parameters in OI children. **(A)** Correlation between serum OC level and glucose metabolic parameters. **(B)** Correlation between serum ucOC level and glucose metabolic parameters. **(C)** Correlation between ratio of ucOC/OC and glucose metabolic parameters. **(D)** Correlation between ratio of OC level and lipid metabolic parameters. **(E)** Correlation between ratio of ucOC level and lipid metabolic parameters. **(F)** Correlation between ratio of ucOC/OC and lipid metabolic parameters.

In OI patients and healthy controls, serum levels of OC, ucOC and ucOC/OC were negatively correlated with BMI, levels of FBG and insulin, and HOMA-IR (**Supplementary Table 2**). Serum levels of OC and ucOC were positively correlated with HDL-C (**Supplementary Table 2**). In OI patients, serum levels of OC, ucOC and ucOC/OC were negatively correlated with BMI, levels of FBG and insulin, and HOMA-IR (**Figure 1**). Serum levels of ucOC and ucOC/OC were negatively correlated with HOMA-β (**Figure 1**). There were no significant correlations between OC, ucOC and ucOC/OC with lipid metabolic parameters (**Figure 1**). Serum OC level was negatively correlated with ambulatory status and fracture frequency, and positively correlated with serum levels of ALP, β-CTX, 25OHD, LS BMD Z-score and FN BMD Z-score. Serum ucOC was negatively correlated with age, ambulatory status and fracture frequency, whereas positively correlated with serum levels of ALP and 25OHD, and LS BMD Z-score (**Supplementary Table 3**).

In multiple linear regression analysis adjusted for age, gender, 25OHD level, eGFR, ambulatory status and clinical classifications, the negative correlation between levels of OC, ucOC and the ratio of ucOC/OC with BMI and serum FBG levels remained (all *P*<0.05, **Table 3**).

**Table 3:** Multiple linear regression analysis to assess correlation between serum OC and ucOC levels with glycolipid metabolic parameters.

	BMI		FBG		Insulin		HOMA-IR		HOMA-β	
	β	*P* value	β	*P* value	β	*P* value	β	*P* value	β	*P* value
OC	-0.260	**0.001**	-0.232	**0.009**	-0.048	0.748	-0.048	0.623	0.056	0.557
ucOC	-0.397	**<0.001**	-0.301	**<0.001**	-0.160	0.089	-0.167	0.078	-0.120	0.196
ucOC/OC	-0.375	**<0.001**	-0.212	**0.017**	-0.154	0.102	-0.151	0.111	-0.161	0.086

OC, osteocalcin; ucOC, undercarboxylated osteocalcin; BMI, body mass index; FBG, fasting blood glucose; HOMA-IR, homeostasis model assessment insulin resistance; HOMA-β, homeostasis model assessment islet beta cell function. Bold values indicate the correlation was significantly different.

This model was adjusted for age, gender, 25OHD, eGFR, ambulatory status and clinical classifications.

### Associations between OC levels with body composition and muscle function

**Figure 2:**
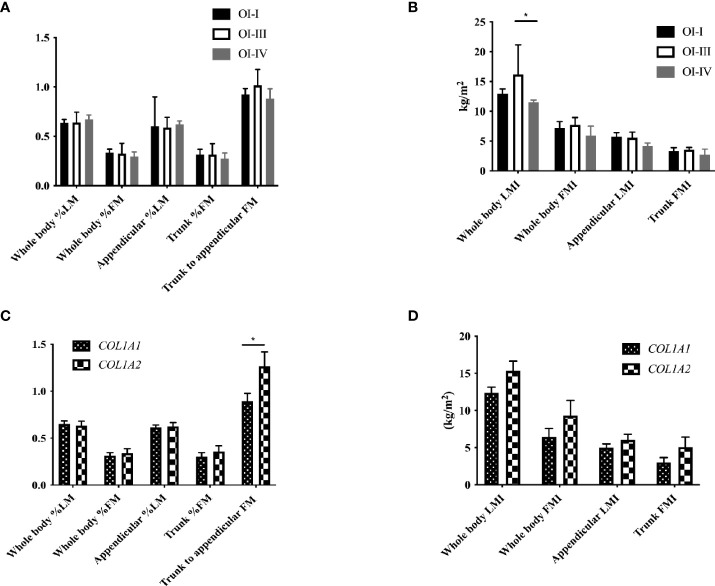
Body composition with different clinical and genotypic classifications of OI children. **(A)** Body composition percentage in OI patients with different clinical classifications. **(B)** Body composition index in OI patients with different clinical classifications. **(C)** Body composition percentage in OI patients with *COL1A1* and *COL1A2* mutation. **(D)** Body composition index in OI patients with *COL1A1* and *COL1A2* mutation. *P < 0.05 for comparison between OI patients with COL1A1 and COL1A2 mutation.

Body composition was measured in 25 OI patients. 14 patients had moderately or severely increased body fat percentage, and 4 patients had slightly increased body fat percentage. The total body LMI of OI type III was significantly lower than that of OI type IV patients (*P*<0.05), and trunk/limb fat mass ratio of the *COL1A2* mutant group was higher than *COL1A1* mutant group (*P*<0.05, **Figure 2**). Serum ucOC level was positively correlated with total body and appendicular %LM (r=0.510, *P*=0.022), but negatively correlated with total body and trunk %FM (r=-0.486, *P*=0.030; r=-0.460, *P*=0.041, respectively). The ratio of ucOC/OC was positively correlated with total body and appendicular %LM (**Supplementary Table 4**; r=-0.463, *P*=0.046). After adjusting for age, gender, 25OHD level, eGFR, ambulatory status and clinical classifications, the correlation between ucOC and body composition still existed (*P*=0.033).

**Figure 3:**
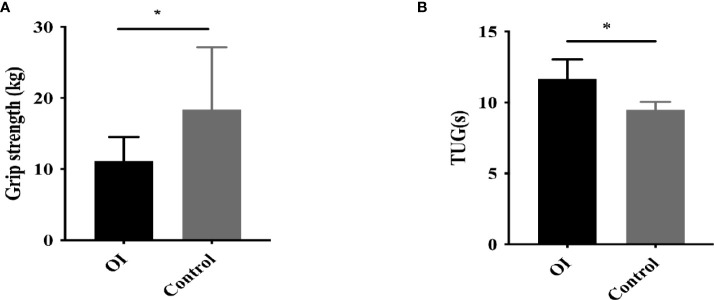
Muscle strength and function in OI patients and healthy controls. **(A)** Grip strength in OI patients and healthy controls. **(B)** Timed-up-and-go (TUG) test in OI patients and healthy controls. *P < 0.05 for comparison between OI patients and COL1A2 healthy controls.

Grip strength and TUG test were measured in 23 and 15 OI patients, as well as 20 healthy controls, respectively. OI patients had significantly lower grip strength and longer TUG than control group (both *P*<0.05, **Figure 3**). Ratio of ucOC/OC was positively correlated with grip strength in OI patients (r=0.512, *P*=0.036).

## Discussion

In this cross-sectional study of relatively large samples of OI patients, we confirmed that overweight or obesity, glucolipid metabolic disorder and impaired muscle function were common in OI children. In OI children, we demonstrated for the first time that circulating levels of OC and ucOC were negatively correlated to impaired glucose metabolism and muscle function. Ratio of ucOC/OC was positively correlated with grip strength in OI patients.

We found that the overweight/obesity was more common in OI children, with higher BMI and body fat percentage than healthy controls, which were closely related to the clinical severity of OI. These results were consistent with the previous studies (9, 21). In a large cohort of OI patients, OI type III patients had higher BMI than type I and type IV OI patients (22). Impaired muscle function was found in our OI patients, which was consistent with weaker muscle force of lower limbs in OI type I children of other studies (23, 24). Patients with OI type I and IV were found to have lower LMI and higher FMI than healthy controls (25). These results all indicated that there was a significant abnormal body composition and muscle strength in OI patients.

Moreover, we found OI children had obvious glycolipid metabolic disorders and insulin resistance, which was previously thought to be caused by reduced movements, less energy expenditure and excessive calorie intake in OI patients (21). In addition, reduced muscle mass in OI was also considered to be caused by insufficient activities. However, we found that there was no obvious correlation between BMI and ambulatory states, and patients with OI type I had less muscle mass and lower grip strength than normal control, though they had similar mobility. Therefore, the ambulatory state could not fully explain the metabolic disorder and muscle dysfunction in OI patients (24, 26, 27), which suggested there may be other mechanisms leading to metabolic and muscular disorder of OI patients.

As we know, OC is the most abundant non-collagen bone matrix protein and has been widely used as a biochemical marker of bone formation. OC is initially synthesized as pre-pro-OC which undergoes proteolytic cleavage to form the mature OC peptide. During bone remodeling, the low pH of resorption lacunae promotes the decarboxylation of OC into ucOC, which reduces its affinity for bone and facilitates its release into the circulation.Carboxylated OC is thought to be predominantly located in bone because of its high binding capacity to hydroxyapatite *in vitro*, whereas ucOC has been reported to function in a paracrine and endocrine manner, participating in glucose metabolism and influencing muscle mass and strength (4, 5). In mouse models, OC and ucOC act as a hormone, with close link to regulation of whole-body metabolism, reproduction, and cognition (28–30). As the active form of circulating OC, ucOC acts on multiple organs to perform endocrine functions, such as pancreas β-cells, adipose, small intestine and skeletal muscle (5). Gprc6a, one putative receptor for osteocalcin, is expressed by pancreas β-cells, adipose and skeletal muscle, which could mediate the effects of osteocalcin on these tissues (31–33). Circulating ucOC could directly activate Gprc6a in islets, thereby triggering islet β-cell proliferation, so as to increase the production and secretion of insulin, which indicated the existence of bone-pancreas loop (34, 35). In adipose tissue, ucOC induced adiponectin secretion, and improved insulin resistance (5, 36). OC also upregulated GLUT4 protein expression in white adipose tissue and promoted insulin-induced phosphorylation of protein kinase B (37). In the small intestine, ucOC indirectly promoted insulin secretion by stimulating glucagon-like peptide-1 (GLP-1) secretion (38). Circulating ucOC enhanced muscle glucose uptake and utilization. Due to the role of OC in different tissues, HOMA-IR appears to have stronger correlations with OC, ucOC and ratio of ucOC/OC than HOMA-β. Studies revealed that lower OC concentrations were associated with higher risk of type 2 diabetes, metabolic syndrome, high BMI and FBG and low insulin sensitivity (39–42). We observed that serum levels of OC were lower in OI patients than those in healthy controls and negatively correlated to BMI, FBG, and HOMA-IR. While in some studies the undercarboxylated form was better correlated with glycometabolic status than carboxylated or total osteocalcin levels (43, 44). Serum ucOC levels and ucOC/OC were negatively correlated to BMI, FBG, HOMA-IR and HOMA-β in OI patients. Previous studies have found that OC levels are different in different genders and changed with age (45). In addition, serum OC and ucOC levels were associated with fracture and vitamin D status (46, 47). Serum OC levels were also affected in patients with renal insufficiency (48). Besides, we found serum OC, ucOC levels, and ucOC/OC were different among patients with different clinical types (**Supplementary Table 1**). Slight differences between OI and controls with regards to ucOC and ratio ucOC/OC may duo to these factors. Therefore, this may be one of the reasons why we observed significant differences in BMI and blood glucose between OI and healthy control, but differences in OC, ucOC and ucOC/OC were not very significant. After adjusting these factors, the correlation sustained. This study found that OI patients had significantly lower HDL-C than healthy controls. As there were no significant correlations between OC, ucOC and ucOC/OC with lipid metabolic parameters, this suggests that there may be other factors except for OC affecting lipid metabolism in OI patients, and it is worthy of further study. These results supported that OC and ucOC may be an important bone derived hormone, which could play essential roles in regulation of glucose metabolism.

Moreover, significantly lower grip strength and longer TUG were found in OI patients than in normal control group, and ratio of ucOC/OC was positively correlated with grip strength, total body and appendicular %LM of OI patients. Gprc6a has also been documented to express in mouse muscle (49, 50). A study indicated that Oc^−/−^ mice had lower muscle mass and average area of the muscle fibers than wild-type mice, and ucOC promoted protein synthesis in myofibers (49). Moreover, exercise was reported to increase circulating interleukin 6 (IL-6) level, which originates from muscle. IL-6 could increase the circulation of ucOC, and promote the uptake and catabolism of glucose and fatty acids in myofibers during exercise in an OC-dependent manner (51). Circulating ucOC enhanced glucose and fatty acid uptake that mainly nourished myofibers, directly or indirectly through IL-6 production (6). OC was necessary to maintain muscle mass in older mice (49). Treatment with ucOC could increase C2C12 myoblasts proliferation and differentiation partly *via* Gprc6a (52). In a cross-sectional study of old women, ucOC/OC was positively correlated with quadriceps muscle strength (53). In our study, serum ucOC level was positively correlated with total body and appendicular lean mass, but negatively correlated with total body and trunk fat mass. Ratio of ucOC/OC was positively correlated with grip strength in OI patients. These studies indicated that ucOC cloud play important roles in regulating muscle mass and muscle function.

OI is a natural model for studying bone fracture. The role of bone in the crosstalk between bone-muscle-pancreas tissue may be better reflected by a natural model of OI. We demonstrated for the first time that OC and ucOC had a close relationship with glycolipid metabolism and muscular function in OI children, which indicated that there may be a close crosstalk between bone-muscle-pancreas-adipose tissue. Ratio of ucOC/OC was positively correlated with grip strength in OI patients, which further confirmed that ucOC can regulate muscle function probably through binding G-protein coupled receptor Gprc6a, and the mutual regulation between skeletal-muscle-fat deserves attention. However, our study had several limitations. First, this study was a cross-sectional study, which was an observational study and could not prove causality. Further in-depth research is needed to elucidate the regulatory mechanism of OC and ucOC in glycolipid metabolism and muscular function of OI patients. Second, the sample size for body composition analysis and glucose tolerance test was quite few. Thirdly, there are many variables included in this study, which will increase the risk of collinearity. However, these variables are correlated with OC to a certain extent, and we have conducted we conducted collinearity analysis among these independent variables and found that there was no collinearity between them. Serum adiponectin secreted by adipose tissue is important to prove the crosstalk between bone-muscle-pancreas-adipose tissue. Serum level of adiponectin was not detected in OI patients, which was also one of the limitations of this study. Last, vitamin K could modulate carboxylation of OC, but we did not measure the vitamin K intake of OI patients.

In conclusion, obesity, glucolipid metabolic abnormalities, and reduced grip strength were common in children with OI. As a bone derived hormone, circulating osteocalcin and ucOC may play an important role in the regulation of glucose metabolism, as well as the muscle function of children with OI. Our results are helpful to reveal the close and interesting crosstalk between bone-muscle-pancreas tissue.

## Data availability statement

The original contributions presented in the study are included in the article/**Supplementary Material**. Further inquiries can be directed to the corresponding author.

## Ethics statement

The studies involving human participants were reviewed and approved by The study was approved by the Scientific Ethics Committee of Peking Union Medical College Hospital. Written informed consent to participate in this study was provided by the participants’ legal guardian/next of kin. Written informed consent was obtained from the minor(s)’ legal guardian/next of kin for the publication of any potentially identifiable images or data included in this article.

## Author contributions

W-BZ carried out the biochemical measurement, collected the clinical data from the patients, analyzed the data and wrote the manuscript. JH, B-NZ and D-CZ contributed to collection of clinical data and blood sample. OW, YJ, W-BX and X-PX contributed to review the manuscript. ML contributed to the conception and design of the research, acquisition and interpretation of the data, and revised the manuscript. All authors contributed to the article and approved the submitted version.

## Funding

This work is supported by National Key R&D Program of China (2018YFA0800801), National Natural Science Foundation of China (No. 81873668, No. 82070908), and Beijing Natural Science Foundation (7202153).

## Acknowledgments

We appreciated the OI patients and normal controls for their participation.

## Conflict of interest

The authors declare that the research was conducted in the absence of any commercial or financial relationships that could be construed as a potential conflict of interest.

## Publisher’s note

All claims expressed in this article are solely those of the authors and do not necessarily represent those of their affiliated organizations, or those of the publisher, the editors and the reviewers. Any product that may be evaluated in this article, or claim that may be made by its manufacturer, is not guaranteed or endorsed by the publisher.
