# Supplementary material for: The role of osteocalcin in regulation of glycolipid metabolism and muscle function in children with osteogenesis imperfecta
Source: Front Endocrinol (Lausanne). 2022 Aug 2;13:898645. doi: 10.3389/fendo.2022.898645 (PMC9378831; doi:10.3389/fendo.2022.898645)
Supplement: Supplementary file 1 [file Table_1.docx]

**Supplementary Table 1. Clinical characteristics of OI patients with different genotypes**

|  | *COL1A1* | *COL1A2* | *IFITM5* | AR | *P* value |
| --- | --- | --- | --- | --- | --- |
|  | (n=126) | (n=66) | (n=8) | (n=22) |  |
| Male, n (%) | 87 (69.0%) | 45 (68.2%) | 3 (37.5%) | 15 (71.4%) | 0.310 |
| Age (y) | 8.0±4.6^b^ | 8.6±4.9^d^ | 3.3±2.3^b,d,f^ | 8.2±4.9^f^ | **0.028** |
| Height (cm) | 122.0±27.3^b^ | 116.6±28.5^d^ | 93.1±16.1^b,d,f^ | 116.7±30.4^f^ | **0.031** |
| Height Z-score | -1.2±2.4^a^ | -2.5±2.6^a^ | -1.0±2.0 | -2.3±2.9 | **0.002** |
| Weight (kg) | 30.0±16.7^b^ | 28.3±15.2^d^ | 15.2±7.7^b,d,f^ | 28.7±16.4^f^ | 0.093 |
| Weight Z-score | 0.0±1.6^a^ | -0.6±1.6^a^ | -0.2±1.8 | -0.5±1.2 | **0.043** |
| BMI (kg/m^2^) | 18.6±3.8 | 19.6±4.2 | 16.7±4.5^c^ | 19.6±4.2^c^ | 0.227 |
| Overweight, n (%) | 9 (7.1%) | 2 (3.0%) | 0 (0) | 1 (4.8%) | 0.066 |
| Obese, n (%) | 22 (17.5%) | 11 (16.7%) | 2 (25.0%) | 6 (28.6%) | 0.076 |
| Nonambulatory, n (%) | 40 (31.7%)^a^ | 32 (48.5%)^a,e^ | 3 (37.5%) | 11 (52.4%)^e^ | 0.078 |
| Times of fracture | 4.0 (3.0, 6.0)^a^ | 5.0 (3.0, 8.0)^a^ | 3.0 (2.0, 5.0) | 4.0 (2.0, 7.8) | 0.057 |
| Ca (mmol/L) | 2.45±0.24 | 2.47±0.12 | 2.52±0.11 | 2.47±0.14 | 0.800 |
| P (mmol/L) | 1.66±0.19^b^ | 1.68±0.24^d^ | 1.50±0.17^b,d^ | 1.62±0.15 | 0.090 |
| ALP (U/L) | 306.2±110.9^a^ | 242.0±79.4^a,d^ | 339.0±168.3^d^ | 282.6±87.3 | **<0.001** |
| β-CTX (ng/ml) | 0.86±0.32^a,c^ | 0.70±0.30^a,e^ | 0.89±0.34 | 1.05±0.52^c,e^ | **0.006** |
| 25OHD (ng/ml) | 22.2 (15.6, 31.0) | 21.8 (15.7, 27.1) | 24.1 (15.1, 35.6) | 28.4 (16.7, 35.2) | 0.901 |
| PTH (ng/ml) | 22.0 (14.0, 31.2) | 21.8 (13.5, 34.2) | 16.9 (12.6, 34.1) | 20.0 (13.0, 45.2) | 0.994 |
| ALT (U/L) | 14.0 (11.0, 18.0) | 14.0 (11.0, 18.0) | 13.5 (10.5, 21.8) | 13.0 (9.3, 22.0) | 0.838 |
| Cr (μmol/L) | 35.9±11.3 | 36.0±14.0 | 30.6±7.9 | 35.9±13.5 | 0.928 |
| eGFR (ml/min/1.73m^2^) | 172.7±40.8 | 171.6±42.0 | 152.5±55.6 | 167.8±42.0 | 0.594 |
| FBG (mmol/L) | 5.02±0.40 | 5.11±0.37 | 5.09±0.61 | 5.20±0.38 | 0.199 |
| Insulin (μIU/mL) | 9.00 (5.30, 16.80) | 9.45 (6.43, 13.05) | 7.00 (4.70, 13.50) | 9.75 (7.63, 15.65) | 0.589 |
| HOMA-IR | 1.93 (1.16, 3.58) | 2.18 (1.44, 3.22) | 1.65 (1.05, 3.48) | 2.25 (1.70, 3.61) | 0.555 |
| HOMA-β (%) | 121.54 (76.58, 191.52) | 113.62 (80.56, 181.54) | 107.50 (77.78, 160.00) | 130.00 (85.63, 212.91) | 0.704 |
| TC (mmol/L) | 4.21±0.75 | 4.23±0.92 | 4.44±0.77 | 4.17±0.65 | 0.668 |
| TG (mmol/L) | 0.82±0.44 | 0.87±0.52 | 0.77±0.39 | 0.73±0.29 | 0.668 |
| HDL-C (mmol/L) | 1.06±0.27 | 1.03±0.23 | 1.11±0.32 | 0.99±0.27 | 0.669 |
| LDL-C (mmol/L) | 2.26±0.55 | 2.22±0.67 | 2.14±0.45 | 2.21±0.40 | 0.925 |
| ucOC (ng/ml) | 11.7±8.4 | 9.7±6.6 | 13.7±9.8 | 11.5±11.2 | 0.351 |
| OC (ng/ml) | 25.1±11.2 | 23.4±10.6 | 27.5±9.3 | 24.7±17.0 | 0.691 |
| ucOC/OC | 0.44±0.19 | 0.39±0.16 | 0.47±0.23 | 0.42±0.21 | 0.488 |
| LS BMD (g/cm^2^) | 0.493±0.193^b^ | 0.443±0.208 | 0.334±0.143^b^ | 0.421±0.201 | 0.051 |
| LS BMD Z-score | -1.9±1.9^a,c^ | -2.6±1.9^a^ | -2.5±2.2 | -3.2±2.4^c^ | **0.013** |
| FN BMD (g/cm^2^) | 0.456±0.183^a^ | 0.378±0.186^a^ | 0.362±0.209 | 0.405±0.220 | **0.036** |
| FN BMD Z-score | -3.3±2.2^a^ | -4.5±2.5^a^ | -4.2±4.6 | -4.1±2.6 | **0.024** |
| Troch BMD (g/cm^2^) | 0.363±0.193 | 0.317±0.174 | 0.250±0.172 | 0.364±0.240 | 0.231 |
| TH BMD (g/cm^2^) | 0.497±0.201 | 0.433±0.199 | 0.380±0.229 | 0.436±0.252 | 0.127 |

AR: autosomal recessive; BMI: body mass index; Ca: calcium; P: phosphate; ALP: alkaline phosphatase; β-CTX: β cross-linked carboxy-terminal telopeptide of type I; 25OHD: 25-hydroxyvitamin D; PTH: parathyroid hormone; FBG: fasting blood glucose; HOMA-IR: homeostasis model assessment insulin resistance; HOMA-β: homeostasis model assessment islet beta cell function; TG: triglyceride; TC: total cholesterol; HDL-C: high density lipoprotein cholesterol; LDL-C: low density lipoprotein cholesterol; OC: osteocalcin; ucOC: undercarboxylated osteocalcin; ALT: alanine aminotransferase; Cr: creatinine; eGFR: estimated glomerular filtration rate. LS: lumbar spine; FN: femoral neck; Troch: trochanter; TH: total hip; BMD: bone mass density.

Bold values indicate that there was a significant difference among 4 groups. ^a^*P*<0.05 for comparison between *COL1A1* and *COL1A2* mutation; ^b^*P*<0.05 for comparison between *COL1A1* and *IFITM5* mutation; ^c^*P*<0.05 for comparison between *COL1A1* mutation and AR group. ^d^*P*<0.05 for comparison between *COL1A2* and *IFITM5* mutation. ^e^*P*<0.05 for comparison between *COL1A2* mutation and AR group. ^f^*P*<0.05 for comparison between *IFITM5* mutation and AR group.

**Supplementary Table 2.** **The simple correlation between serum levels of OC and ucOC and with glycolipid metabolic parameters in all subjects**

|  | OC | |  | ucOC | |  | ucOC/OC | |
| --- | --- | --- | --- | --- | --- | --- | --- | --- |
|  | r | *P* value |  | r | *P* value |  | r | *P* value |
| BMI | -0.178 | **0.003** |  | -0.252 | **<0.001** |  | -0.153 | **0.012** |
| FBG | -0.239 | **<0.001** |  | -0.212 | **<0.001** |  | -0.139 | **0.025** |
| Insulin | -0.131 | **0.045** |  | -0.213 | **<0.001** |  | -0.161 | **0.014** |
| HOMA-IR | -0.155 | **0.018** |  | -0.231 | **<0.001** |  | -0.164 | **0.012** |
| HOMA-β | -0.025 | 0.709 |  | -0.110 | 0.086 |  | -0.108 | 0.099 |
| TC | 0.078 | 0.235 |  | 0.120 | 0.059 |  | 0.090 | 0.171 |
| LDL-C | -0.001 | 0.990 |  | 0.061 | 0.340 |  | 0.096 | 0.144 |
| HDL-C | 0.166 | **0.011** |  | 0.196 | **0.002** |  | 0.077 | 0.240 |
| TG | 0.008 | 0.902 |  | 0.048 | 0.448 |  | 0.103 | 0.117 |

OC: osteocalcin; ucOC: undercarboxylated osteocalcin; BMI: body mass index; FBG: fasting blood glucose; HOMA-IR: homeostasis model assessment insulin resistance; HOMA-β: homeostasis model assessment islet beta cell function; TG: triglyceride; TC: total cholesterol; HDL-C: high density lipoprotein cholesterol; LDL-C: low density lipoprotein cholesterol; 25OHD: 25-hydroxyvitamin D; Bold values indicate the correlation was significantly different.

**Supplementary Table 3.** **The simple correlation between serum levels of OC and ucOC and phenotypes of OI patients**

|  | OC | |  | ucOC | |  | ucOC/OC | |
| --- | --- | --- | --- | --- | --- | --- | --- | --- |
|  | r | *P* value |  | r | *P* value |  | r | *P* value |
| Gender | 0.028 | 0.676 |  | 0.084 | 0.214 |  | 0.013 | 0.845 |
| Age | -0.234 | **<0.001** |  | -0.214 | **0.001** |  | -0.135 | **0.046** |
| BMI | -0.399 | **<0.001** |  | -0.549 | **<0.001** |  | -0.454 | **<0.001** |
| FBG | -0.315 | **<0.001** |  | -0.322 | **<0.001** |  | -0.222 | **0.001** |
| Insulin | -0.201 | **0.006** |  | -0.280 | **<0.001** |  | -0.228 | **0.002** |
| HOMA-IR | -0.233 | **0.002** |  | -0.307 | **<0.001** |  | -0.237 | **0.001** |
| HOMA-β | -0.085 | 0.256 |  | -0.149 | **0.044** |  | -0.150 | **0.045** |
| TC | 0.076 | 0.305 |  | 0.115 | 0.119 |  | 0.086 | 0.248 |
| LDL-C | -0.024 | 0.742 |  | 0.047 | 0.524 |  | 0.009 | 0.225 |
| HDL-C | 0.140 | 0.057 |  | 0.133 | 0.069 |  | 0.055 | 0.460 |
| TG | -0.024 | 0.742 |  | 0.040 | 0.588 |  | 0.097 | 0.190 |
| Ambulatory status | -0.248 | **<0.001** |  | -0.179 | **0.008** |  | -0.051 | 0.452 |
| Clinical classification | -0.023 | 0.737 |  | 0.041 | 0.540 |  | 0.074 | 0.276 |
| Times of fracture | -0.237 | **0.001** |  | -0.175 | **0.009** |  | -0.068 | 0.321 |
| ALP | 0.271 | **<0.001** |  | 0.217 | **0.001** |  | 0.130 | 0.057 |
| β-CTX | 0.168 | **0.032** |  | 0.145 | 0.065 |  | 0.055 | 0.485 |
| 25OHD | 0.281 | **0.001** |  | 0.234 | **0.004** |  | 0.026 | 0.756 |
| PTH | 0.110 | 0.161 |  | 0.123 | 0.118 |  | 0.085 | 0.284 |
| LS BMD | 0.052 | 0.442 |  | 0.006 | 0.928 |  | -0.048 | 0.486 |
| FN BMD | 0.053 | 0.438 |  | -0.009 | 0.893 |  | -0.066 | 0.331 |
| Troch BMD | -0.055 | 0.426 |  | -0.083 | 0.229 |  | -0.081 | 0.245 |
| TH BMD | -0.008 | 0.904 |  | -0.074 | 0.292 |  | -0.118 | 0.092 |
| LS BMD Z-score | 0.258 | **<0.001** |  | 0.152 | **0.024** |  | 0.023 | 0.723 |
| FN BMD Z-score | 0.136 | **0.044** |  | 0.028 | 0.675 |  | -0.034 | 0.618 |
| Cr | -0.124 | 0.071 |  | -0.114 | 0.099 |  | -0.073 | 0.295 |
| eGFR | 0.086 | 0.215 |  | 0.066 | 0.342 |  | 0.087 | 0.209 |

OC: osteocalcin; ucOC: undercarboxylated osteocalcin; BMI: body mass index; FBG: fasting blood glucose; HOMA-IR: homeostasis model assessment insulin resistance; HOMA-β: homeostasis model assessment islet beta cell function; TG: triglyceride; TC: total cholesterol; HDL-C: high density lipoprotein cholesterol; LDL-C: low density lipoprotein cholesterol; ALP: alkaline phosphatase; 25OHD: 25-hydroxyvitamin D; PTH: parathyroid hormone; LS: lumbar spine; FN: femoral neck; Troch: trochanter; TH: total hip; BMD: bone mass density. Cr: creatinine; eGFR: estimated glomerular filtration rate. Bold values indicate the correlation was significantly different.

**Supplementary Table 4. The simple correlation between serum levels of OC and ucOC with muscle parameters and body composition of OI patients**

|  | OC | |  | ucOC | |  | ucOC/OC | |
| --- | --- | --- | --- | --- | --- | --- | --- | --- |
|  | r | *P* value |  | r | *P* value |  | r | *P* value |
| Grip strength | 0.073 | 0.772 |  | -0.809 | 0.096 |  | 0.465 | 0.060 |
| TUG | 0.030 | 0.934 |  | -0.015 | 0.942 |  | -0.100 | 0.798 |
| Total body LM (kg) | 0.129 | 0.587 |  | 0.026 | 0.915 |  | 0.015 | 0.955 |
| Total body LM% | 0.268 | 0.254 |  | 0.510 | **0.022** |  | 0.463 | **0.046** |
| Total body LMI | 0.042 | 0.860 |  | 0.218 | 0.433 |  | 0.367 | 0.123 |
| Appendicular LM (kg) | 0.033 | 0.890 |  | -0.116 | 0.627 |  | -0.135 | 0.581 |
| Appendicular LM (%） | 0.224 | 0.342 |  | 0.556 | **0.011** |  | 0.567 | **0.011** |
| Appendicular LMI | 0.223 | 0.346 |  | 0.155 | 0.514 |  | 0.086 | 0.726 |
| Total body FM (kg) | -0.211 | 0.373 |  | -0.367 | 0.112 |  | -0.274 | 0.257 |
| Total body FM% | -0.227 | 0.336 |  | -0.486 | **0.030** |  | -0.432 | 0.065 |
| Total body FMI | -0.126 | 0.596 |  | -0.311 | 0.182 |  | -0.270 | 0.263 |
| Trunk FM (kg) | -0.165 | 0.486 |  | -0.323 | 0.164 |  | -0.228 | 0.348 |
| Trunk FM% | -0.217 | 0.359 |  | -0.460 | **0.041** |  | -0.416 | 0.077 |
| Trunk FMI | -0.119 | 0.618 |  | -0.308 | 0.186 |  | -0.263 | 0.276 |
| Trunk/limb fat mass ratio | -0.053 | 0.823 |  | -0.035 | 0.882 |  | 0.108 | 0.660 |

OC: osteocalcin; ucOC: undercarboxylated osteocalcin; TUG: timed-up-and-go; LM: lean mass; LMI: 1ean mass index; FM: fat mass; FMI: fat mass index. Bold values indicate the correlation was significantly different.
